# A Case of Herpes Zoster Ophthalmicus With Multiple Delayed Ocular Complications

**DOI:** 10.7759/cureus.37134

**Published:** 2023-04-04

**Authors:** Yasuyuki Takai, Akiko Yamagami, Mayumi Iwasa, Kenji Inoue, Masato Wakakura

**Affiliations:** 1 Ophthalmology, Inouye Eye Hospital, Tokyo, JPN

**Keywords:** intraorbital inflammation, dacryoadenitis, optic perineuritis, varicella-zoster virus, herpes zoster ophthalmicus

## Abstract

Herpes zoster ophthalmicus (HZO) presents a variety of ocular complications, most of which occur simultaneously as skin lesions. We report a case of HZO with delayed onset of multiple ocular complications. A 72-year-old man developed HZO, blepharitis, iritis, and conjunctivitis in the left eye, which resolved after topical ocular treatment and systemic acyclovir administration. However, six weeks after the first onset of the rash, the patient came to our hospital because of recurrent blepharitis, iritis, scleritis, conjunctivitis, eye pain, ptosis, and blurred vision in the left eye. Best corrected visual acuity (BCVA) in the left eye had decreased to hand motion, and the Goldmann visual field test showed only mild residual peripheral vision on the lateral side. Intraocular pressure showed 25 mmHg in the left eye and inflammation in the anterior chamber with paralytic mydriasis. Orbital magnetic resonance imaging (MRI) showed the contrast effects with the lacrimal gland, superior ophthalmic vein, supraorbital nerve, optic nerve, and around optic nerve sheath. The patient was diagnosed with optic neuritis, optic perineuritis, ptosis, paralytic mydriasis, trigeminal neuralgia, lacrimal gland inflammation, blepharitis, iritis, scleritis, and ocular hypertension after HZO, and three courses of steroid pulse therapy were administered. Thereafter, BCVA improved to 0.3 in the left eye, with improvement in central vision, and MRI lesions and other symptoms also improved. The patient has had no complications or recurrence of HZO. HZO can cause a variety of ocular complications. Since autoimmune mechanisms might be involved, combined immunotherapy should be considered.

## Introduction

Herpes zoster ophthalmicus (HZO) is caused by the reactivation of the varicella-zoster virus (VZV) along the first (ophthalmic) branch of the trigeminal nerve [1]. It occurs in one-quarter of cases of herpes zoster and presents as a periorbital vesicular rash along the affected skin segment. Since the trigeminal nerve provides sensory nerve supply to the eye, it can also cause various ocular complications. The ocular lesions are intractable neuropathic pain, conjunctivitis, keratitis, uveitis, and ocular muscle paralysis, and the complications occur shortly after the onset of skin rash [1]. In this report, we describe a case of herpes zoster ophthalmicus with delayed onset of a variety of ocular complications.

## Case presentation

A 72-year-old man with a history of normal tension glaucoma developed HZO of the left eye. He was treated with an intravenous infusion of acyclovir, topical steroids (0.1% betamethasone), and acyclovir ophthalmic ointment, and the symptoms improved after two weeks of onset. Four weeks after the improvement of HZO, the patient became aware of visual loss in the left eye, and blepharitis, ptosis, pain around the periocular region, iritis, and scleritis were observed in the left eye. Hence, the patient was referred to our hospital six weeks after the onset of the skin rash.

**Figure 1:**
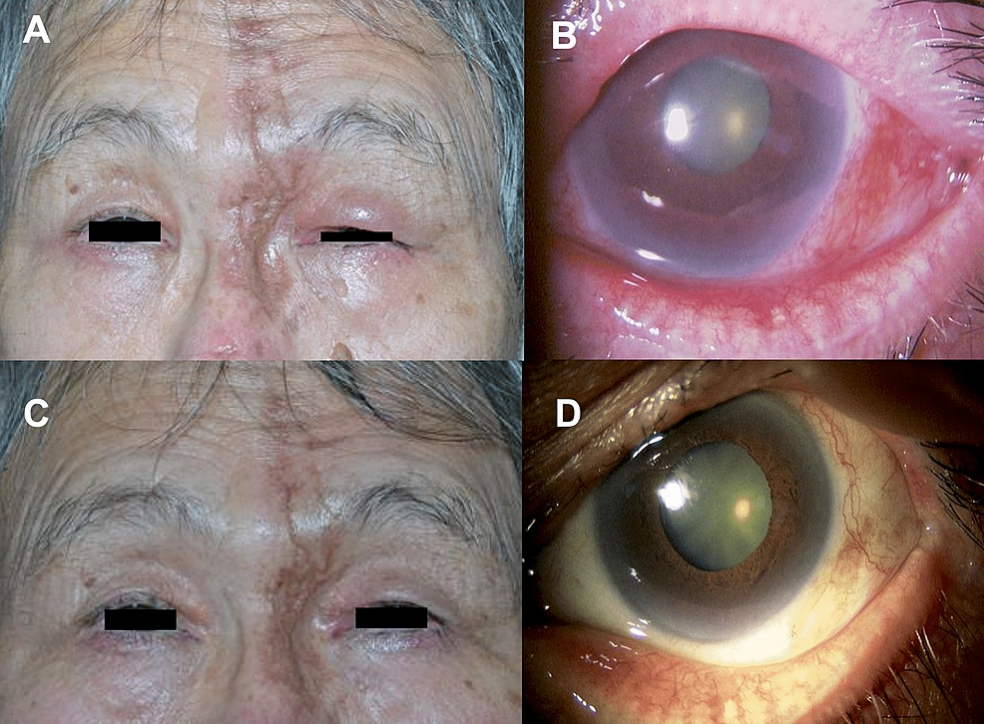
External and anterior ocular segment at the initial visit and after treatment with one course of steroid semi-pulses therapy External ocular evaluation at the initial visit indicated blepharitis of the left eye with concomitant ptosis (A, B). The slit-lamp examination showed conjunctivitis and scleritis with intra-orbital inflammation (B). After treatment, blepharitis and ptosis resolved (C), and the anterior segment findings improved (D).

**Figure 2:**
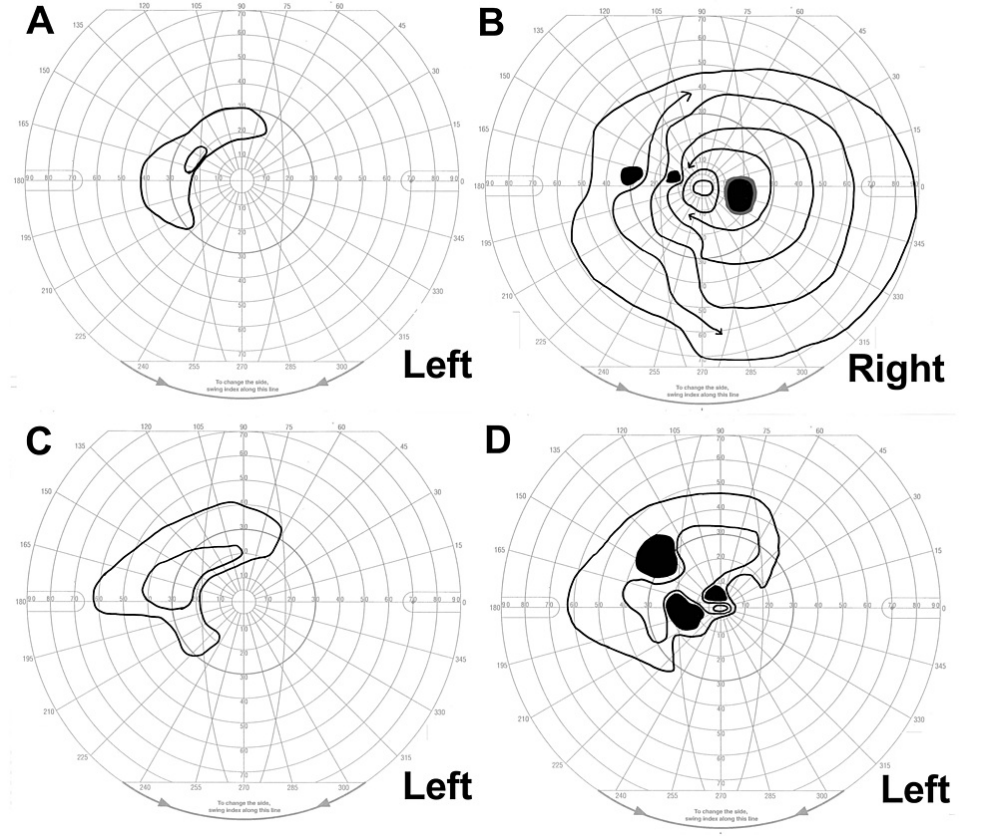
Goldmann visual field test findings at the initial visit and after treatment with steroid pulse therapy At the initial examination, the left eye had only nasal peripheral vision (A). The right eye showed a paracentral dark spot due to glaucoma (B). After one course of steroid-pulse therapy, the peripheral vision expanded (C) and, after three courses of steroid-pulse therapy, the peripheral vision expanded further, along with recovery of central vision (D).

**Figure 3:**
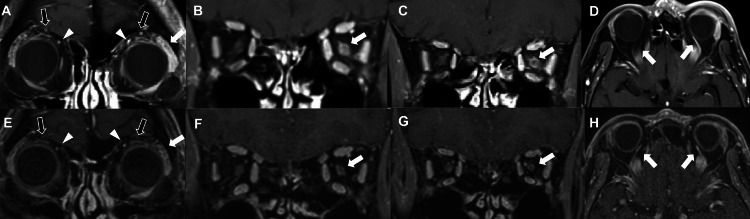
Orbital MRI findings at initial examination and after treatment with 3 courses of steroid pulses (Gd contrast T1-weighted image) Orbital MRI at the initial visit showed the high-intensity lesion of an enlarged lacrimal gland (A, white arrow), and of the mildly dilated superior ophthalmic vein (A. white arrowhead) and supraorbital nerve (A, black arrow) in the left orbital. The optic nerve of the left eye showed a contrast effect, indicating optic neuritis (B, white arrow), which was complicated with a contrast effect around the optic nerve sheath (C, white arrow). Axial section images also showed significant high signal intensity and mild dilation of the superior ophthalmic vein on the left side (D, white arrow). On orbital MRI after treatment, the lacrimal gland enlargement and high-intensity lesion in the left orbit were reduced (E, white arrow), and the left-right difference in the high-intensity lesion of the superior ophthalmic vein (E, white arrowhead) and supraorbital nerve (E, black arrow) was also less noticeable. The contrast effect on the optic nerve and optic nerve sneath also disappeared (F and G, arrowheads).

Best corrected visual acuity (BCVA) was 20/16 in the right eye and hand motion in the left eye, and intraocular pressure (IOP) was 12 mmHg in the right eye and 25 mmHg in the left eye (Goldmann applanation tonometer). There was no active skin rash. The left eyelids were erythematous and swollen (Figure 1A), and ptosis and periocular pain were also noted, with no ocular motility disturbances. The pupil was dilated to 4.5 mm in the left eye, and the direct and indirect light reflex in the left eye had disappeared. Additionally, the left eye had developed conjunctivitis and scleritis, and inflammatory cells in the anterior chamber were also observed (Figure 1B). Fundus examination revealed no findings other than bilateral optic disc cupping for glaucoma. The Goldmann visual field test showed that only temporal peripheral vision remained in the left eye (Figure 2A), along with the presence of paracentral dark spots due to glaucoma in the right eye (Figure 2B). Orbital magnetic resonance imaging (MRI) with post-contrast T1-weighted imaging showed the high-intensity lesion of an enlarged lacrimal gland (Figure 3A) and the mild dilation of the superior ophthalmic vein (Figure 3A, D) and supraorbital nerve (Figure 3A) in the left orbit. Orbital MRI also revealed optic neuritis and optic perineuritis (Figure 3B, C). The orbital apex and cavernous sinus were unaffected, and there were no intracerebral lesions involving the oculomotor or trigeminal nuclei. Blood tests showed no systemic inflammatory reaction and no findings suggestive of autoimmune disease.

The diagnosis was left optic neuritis, optic perineuritis, ptosis, paralytic mydriasis, trigeminal neuralgia, lacrimal gland inflammation, blepharitis, iritis, scleritis, and ocular hypertension after HZO. Topical steroids (0.1% betamethasone) and acyclovir ophthalmic ointment alone did not improve his symptoms, including anterior segment findings, and he was treated with steroid semi-pulse therapy (methylprednisolone 500 mg/day for three days). Systemic acyclovir administration was not concomitantly administered because the patient was on antiviral therapy with a sufficient dose of acyclovir at the time of initial onset, and there was no active herpes zoster infection. At the end of the first steroid therapy, BCVA was 6/600 in the left eye, and the Goldmann visual field test showed mild improvement in peripheral vision (Figure 2C). Eyelid swelling, ptosis, periocular pain, iritis, and conjunctival and scleritis improved markedly, and intraocular pressure normalized to 14 mmHg in the left eye. There were no findings of reactivation of the herpes zoster virus. Hence, two additional courses of steroid pulse therapy (methylprednisolone 1000 mg/day for three days) were added. At the end of treatment, BCVA in the left eye had improved to 6/30, and Goldmann visual field testing showed that the peripheral visual field was enlarged and a central visual field was recognized (Figure 2D). On orbital MRI after the third steroid-pulse course, lacrimal gland inflammation, optic neuritis, and optic perineuritis improved on the gadolinium contrast T1-weighted image (Figure 3E, F, G). The high-intensity lesion of the superior ophthalmic vein (Figure 3E, H) and the supraorbital nerve (Figure 3E) with mild dilation were also reduced, and the left-right difference became less noticeable. Three months after the onset of the disease, BCVA in the left eye had improved to 6/20. Since then, the patient has been followed up without treatment and has had no recurrence or other complications due to herpes zoster for 10 months.

## Discussion

Herpes zoster ophthalmicus (HZO) occurs in 10-25% of herpes zoster cases [1], and it is estimated that 50% of these cases have ocular complications [2]. The ocular complications are diverse, with keratitis being the main complication, occurring in 76.2% of cases, with iritis/uveitis in 46.6%, conjunctivitis in 35.4%, and scleritis in 10.6% of patients [3]. Neuro-ophthalmologic complications are even rarer but can include intraorbital inflammation, such as lacrimal gland inflammation, orbital apex syndrome, optic peri neuritis, and extraocular myositis, as well as cranial nerve palsies, such as blepharoptosis, eye movement disorder, and optic neuritis [2]. The present case also had a variety of complications, including optic neuritis, optic perineuritis, blepharoptosis, paralyzing mydriasis, trigeminal neuralgia, lacrimal gland inflammation, blepharitis, iritis, scleritis, and ocular hypertension. Intraorbital complications were reported to occur more frequently around 10 days after the onset of skin rash in a study of 21 cases of orbital apex syndrome with HZO and are often seen during the acute phase of VZV infection [4]. The present case showed delayed ocular complications after six weeks from the appearance of a skin rash associated with HZO.

In the present case, MRI showed reversible lesions of the superior ophthalmic vein and the supraorbital nerve, a branch of the trigeminal nerve. Pintwata et al. reported the superior ocular vein dilation associated with ocular zoster [5]. The possible mechanisms were considered as thrombus formation associated with vasculitis obliterans and stasis of blood flow associated with intraorbital inflammation. Although MRI findings of trigeminal neuropathy associated with herpes zoster are reported, many cases have shown high signal intensity in the trigeminal nucleus and trigeminal ganglion [6,7]. Abnormal MRI findings of the supraorbital nerve are often reported in neoplastic diseases such as schwannoma [8,9], but there are no reports of such reversible supraorbital nerve lesions in the HZO. When intraorbital inflammation is seen, detailed MRI findings may be useful in the diagnosis.

As for other cranial nerve deficits, the patient was accompanied by mydriasis and ptosis in the left eye, suggesting a disturbance of the oculomotor nerve. There were no findings on head MRI to suggest damage to the oculomotor nucleus or intramedullary fibers in the midbrain, the cavernous sinus, or the oculomotor nerve at the orbital apex and no evidence of ocular movement disorder. Paralytic mydriasis has been reported after herpes zoster infection [10,11], and ischemic damage associated with vasculitis obliterans of the pupillary sphincter or peripheral branches of the oculomotor nerve has been implicated as a possible mechanism. There have been reports of ptosis without other oculomotor neuropathies, suggesting a mechanism of injury to the peripheral branches of the oculomotor nerve [12]. HZO can result in an isolated injury to a peripheral branch or its innervating muscles, and this should be recognized as a complication.

In the present case, the patient developed delayed ocular complications, as well as intraorbital inflammation and cranial nerve palsy. Other than paralytic mydriasis, the complications improved only with systemic administration of steroids without concomitant antiviral therapy. The route of entry of the virus into each tissue includes direct entry of the virus trans-synaptically and trans-hematologically, spillover from the meninges and brain tissue [13]​​​​​​​. The mechanisms of tissue damage associated with VZV infection include direct damage by the virus itself, indirect damage by the immune response, and ischemia due to vasculitis [14]. In the present case, considering the use of a sufficient amount of acyclovir in the acute phase and the possibility of a strong involvement of immunological mechanisms, systemic administration of acyclovir was not used at the time of relapse, and systemic administration of steroids resulted in marked improvement, including of local ocular symptoms. Thus, delayed ocular complications may develop due primarily to autoimmune inflammation.

The delayed onset of ocular complications of VZV may be influenced by the excessive autoimmune inflammation against VZV. Gliden et al. have examined the presence of VZV antigens in temporal artery specimens obtained by temporal artery biopsy in giant cell arteritis cases and normal (autopsy) cases [15]. In giant cell arteritis, the VZV antigen was present in as many as 70% of cases, compared with 18% of controls. Most of the VZV antigens were found in accordance with the skip lesions and were concentrated in the outer membrane of the vessels, where there are many afferent nerve fibers. Given the fact that giant cell arteritis is treated with immunosuppressive therapies such as steroids [16], it is possible that an excessive immune response to attenuated VZV antigen might be the cause of the inflammation in that condition. The VZV infection as a prior infection was followed by the development of neuro-autoimmune diseases, such as Guillain-Barré syndrome [17]​​​​​​​​​​​​​​ and acute disseminated encephalomyelitis [18], suggesting the involvement of an autoimmune mechanism as a delayed complication of VZV infection.

Tran et al. reported chronic and recurrent ocular complications associated with VZV and showed that one-fourth of cases could replace for five years [19]. Conjunctivitis and scleritis were more common at the initial onset, whereas keratitis and uveitis were more common at recurrence. Although details of the mechanisms are unknown, both viral and immune-mediated involvement have been postulated. In this case, the patient had a delayed onset of iritis, conjunctivitis, scleritis, and ocular hypertension. The systemic administration of steroids had been significantly effective against it, so an immune-mediated mechanism was assumed for this case. Previously, a pathological examination of an excised eye with HZO showed chronic inflammatory cell infiltration of the long posterior ciliary artery and nerves [20]. A case of uveitis after vaccination with an inactivated recombinant herpes zoster vaccine has also been reported [21]. It can be said that inflammation due to immune response may be stronger in delayed ocular complications than the direct damage by VZV itself.

Treatment of intraorbital inflammation associated with herpes zoster ophthalmicus consists of systemic acyclovir in the acute phase of the infection. Verhaeghe et al. reported 15 cases of orbital apex syndrome caused by HZO, including their own case, and in all cases, systemic steroids were administered in addition to systemic acyclovir [13]. In their own case, the patient treated with systemic acyclovir alone had exacerbations and improved after concomitant steroid therapy. In addition, a systematic review of the treatment of Ramsey-Hunt syndrome, a typical neurologic complication of VZV, recommends anti-inflammatory treatment with steroids [22]. The present case is a single case report, and the spinal fluid findings were not confirmed. Therefore, the evidence is not sufficient to recommend steroid monotherapy for delayed complications, but the combination of steroids should at least be considered early in the course of the disease, including this evidence of treatment in the acute phase of intraorbital inflammation and cranial nerve dysfunctions, as immunological mechanisms may be particularly conspicuous in delayed-onset cases.

## Conclusions

HZO can cause various complications, not only ocular complications but also intraorbital inflammation and cranial nerve palsy. It can occur not only in the acute phase of infection but also in the delayed phase. In the case of delayed onset, immunological mechanisms may be strongly involved, and combination immunotherapy should be considered.
